# Invariance and optimality in the regulation of an enzyme

**DOI:** 10.1186/1745-6150-8-7

**Published:** 2013-03-22

**Authors:** Ed Reznik, Stefan Yohe, Daniel Segrè

**Affiliations:** 1 Department of Biomedical Engineering, Boston University, 44 Cummington Mall, Boston, MA 02215, USA; 2 Bioinformatics Program and Department of Biology, Boston University, 44 Cummington Mall, Boston, MA 02215, USA

## Abstract

**Background:**

The Michaelis-Menten equation, proposed a century ago, describes the kinetics of enzyme-catalyzed biochemical reactions. Since then, this equation has been used in countless, increasingly complex models of cellular metabolism, often including time-dependent enzyme levels. However, even for a single reaction, there remains a fundamental disconnect between our understanding of the reaction kinetics, and the regulation of that reaction through changes in the abundance of active enzyme.

**Results:**

We revisit the Michaelis-Menten equation under the assumption of a time-dependent enzyme concentration. We show that all temporal enzyme profiles with the same average enzyme level yield identical substrate degradation– a simple analytical conclusion that can be thought of as an invariance principle, and which we validate experimentally using a β-galactosidase assay. The ensemble of all time-dependent enzyme trajectories with the same average concentration constitutes a space of functions. We develop a simple model of biological fitness which assigns a cost to each of these trajectories (in the form of a function of functions, *i.e*. a functional). We then show how one can use variational calculus to analytically infer temporal enzyme profiles that minimize the overall enzyme cost. In particular, by separately treating the static costs of amino acid sequestration and the dynamic costs of protein production, we identify a fundamental cellular tradeoff.

**Conclusions:**

The overall metabolic outcome of a reaction described by Michaelis-Menten kinetics is ultimately determined by the average concentration of the enzyme during a given time interval. This invariance in analogy to path-independent phenomena in physics, suggests a new way in which variational calculus can be employed to address biological questions. Together, our results point to possible avenues for a unified approach to studying metabolism and its regulation.

**Reviewers:**

This article was reviewed by Sergei Maslov, William Hlavacek and Daniel Kahn.

## Background

Cells regulate enzyme levels and activities to address specific metabolic demands. Despite the availability of large metabolic and transcriptional data sets, understanding the logic and dynamics of metabolic regulation remains a fundamental ongoing challenge [1-3]. This is partly due to the fact that metabolic regulation depends on the complex interplay between transcription, translation, post-translational modifications, and feedback from metabolism itself [3,4]. While significant strides in this field are increasingly being made at the level of genome-scale models of metabolic and regulatory networks [5,6], one may wonder whether new insight can still be found in the study of individual biochemical reactions. To this end, we investigate how the rate of a biochemical reaction is affected by the control and modification of its crucial component, the catalyzing enzyme. We do so by re-examining the classical Michaelis-Menten (MM) enzyme catalysis mechanism in conditions where the total enzyme concentration changes over time.

While enzyme levels can change dramatically throughout the lifetime of a cell, most mathematical models of metabolism in which MM equations are used assume constant enzyme levels. This assumption is often justified by the fact that enzyme levels change much slower than the typical relaxation times of metabolic network dynamics. Importantly, a number of authors have addressed the question of how the MM equation would be modified, under different assumptions of a time-dependent enzyme concentration (for example, the degradation of enzyme) [7-15]. In addition, models simultaneously accounting for enzyme kinetics, enzyme protein synthesis/degradation and genetic regulation have been constructed for a number of specific biochemical networks (for example, see [16-19]). However, a deep understanding of the complex interplay between these levels of biological organization still represents a fundamental challenge.

In this work we show how certain properties of a biochemical reaction with time-dependent enzyme concentration can be summarized and explored through a very simple analytical re-elaboration of the classical MM equation. In particular, we show that the apparent diversity of behaviors ensuing from different enzyme trajectories can be reduced to the extreme simplicity of an invariance principle. This principle analytically relates the amount of substrate consumed in a given period of time to the average enzyme level during that interval, irrespective of the specific enzyme level trajectory. We analytically illustrate how this simple dependence may be extended to more complex single-enzyme reactions, and experimentally verify the invariance to average enzyme levels using a β-galactosidase assay. In the final section, using variational calculus, we apply this invariance principle to ask whether specific enzyme trajectories may be optimally advantageous to the cell under different assumptions on the relative costs associated with producing large amounts of enzyme slowly, versus producing modest amounts of enzyme quickly. Using classical concepts from optimal control theory and variational calculus, we show how such questions may be answered analytically.

## Results

### Michaelis-Menten kinetics depends on average enzyme levels

Consider an enzymatically-catalyzed biochemical reaction, in which a substrate *S* is degraded to a product *P* through the action of an enzyme *E*:

(1) $$E_{U}+S\overset{k_{1}}{\to }\underset{k_{-1}}{\leftarrow }C\overset{k_{2}}{\to }P+E_{U}$$

When the total concentration of enzyme *E = E*_*U*_*+ C* (the sum of unbound enzyme *E*_*U*_ and enzyme-substrate complex *C*) remains low throughout the course of the reaction (*i.e. E << K*_*M*_*+ S*, where *K*_*M*_ = (*k*_-1_*+k*_2_)/*k*_1_), Michaelis-Menten (MM) kinetics can be used to model the reaction (*i.e.* by applying the quasi-steady-state assumption, see [20]). We further assume that *E* changes at a characteristic rate that is much slower than the rapid equilibration of *C*, but we do not assume that it is constant. Instead, as is often done in modeling of hybrid metabolic-genetic circuits [21,22], we explicitly treat the concentration of enzyme as a function of time, *E*(*t*). Then, the resulting dynamics of *S* can be described by the modified MM equation:

(2) $$\frac{\mathrm{dS}}{\mathrm{dt}}=-\frac{k_{2}E\left(t\right)S}{K_{M}+S}$$

with initial conditions *E*(*t*_*0*_) = *E*_*0*_, *S*(*t*_*0*_) = *S*_*0*_. The main question we ask is: how does substrate dynamics depend on the details of *E*(*t*)? To answer this question, we rearrange Equation (2), so as to isolate *S*(*t*) and *E*(*t*) on opposite sides of the equation:

(3) $$\frac{K_{M}+S}{k_{2}S}\mathrm{dS}=-E\left(t\right)\mathrm{dt}\;$$

This equation can be easily integrated between *t=t*_*0*_ and *t=t*_*f*_, giving rise to

(4) $$-\frac{1}{k_{2}\Delta t}\left(K_{M}\ln\frac{S_{f}}{S_{0}}+S_{f}-S_{0}\right)=\bar{E}$$

where *S*_*f*_ = *S*(*t*_*f*_), *Δt* = *t*_*f*_ – *t*_*0*_, and $\bar{E}=\frac{1}{\mathrm{\Delta t}}\int_{t_{o}}^{t_{f}}E\left(t\right)\mathrm{dt}$. The right hand side of this equation $\left(\bar{E}\right)$ represents the average amount of enzyme in the system during the time interval *Δt*, and the left hand side is a monotonic function of the boundary conditions for *S.*

Eq. (4) states that *given an initial amount of substrate, the amount of substrate consumed during a given time interval Δt depends only on the average enzyme level during that interval, and not on the specific, time-dependent enzyme trajectory*. Any two time-course profiles of *E* encountering the same initial substrate concentration *S*_*0*_ and exhibiting identical average amounts of enzyme $\bar{E}$ will degrade precisely the same amount of substrate in a given time interval. The temporal dynamics of the enzyme does not play any role in the final concentration of substrate: the metabolic process is solely a function of the average enzyme level.

It is worth noting that the dependence of substrate kinetics on *average* enzyme levels also holds, in a modified form, for more complex biochemical reactions catalyzed by a single enzyme. In the SI text, we explicitly present two such cases. First, we consider a bisubstrate reaction, in which two substrates are converted to a product. Second, we consider the case where two substrates may be simultaneously competing for the active site of a single enzyme. Such competition for the enzymatic active site plays important roles in the behavior of metabolic pathways [23] as well as synthetic biological circuits [24]. Although in both cases the formula relating the boundary conditions of substrate and average enzyme levels is more complicated, it remains true that the average enzyme level entirely determines the final concentration of the substrates given their initial concentration. These two results highlight the key feature which renders MM kinetics (and a broad category of other biochemical rate laws) invariant to time-dependent enzyme profiles with the same average enzyme concentration: the separability of the rate law into terms corresponding exclusively to enzyme and substrate. In other words, it must be possible for the rate law to be written as *dS*/*dt* = *E* ∙ *f*(*S*). While it is unlikely that this dependency on average enzyme levels will directly generalize to larger networks of metabolic reactions (where such separability is in general no longer possible), the notion of finding and exploiting conserved dynamical quantities in such systems may be a prominent route for future work.

As far as we can tell, the invariance of MM kinetics to the time-dependent details of enzyme trajectories with identical average concentrations seems not to have been explored in the enzyme kinetics and computational biology literature, despite its simplicity. In the next section, we describe an experiment aimed at verifying the validity of this analytically derived result. Then, we illustrate how this invariance principle may be used to probe the nature of metabolic regulation.

### Experimental validation

**Figure 1:**
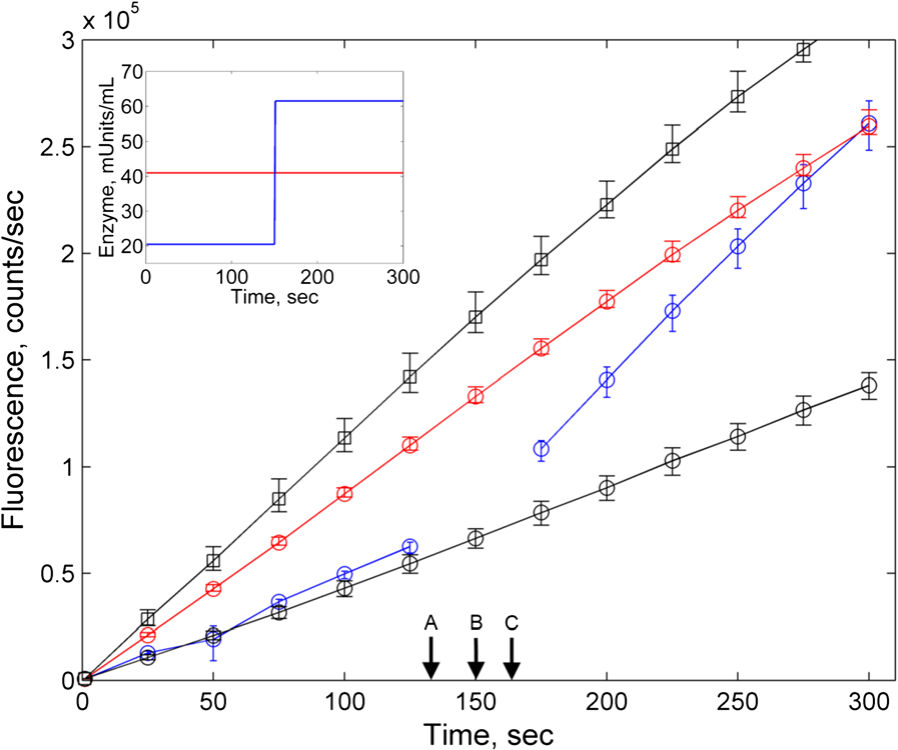
**Results from the β-galactosidase enzymatic assay, aimed at testing the metabolic equivalence of different enzyme trajectories.** Red lines correspond to the constant enzyme experiment, blue lines correspond to the enzyme step-function experiment. The main figure shows the level of fluorescence due to the concentration of reaction product, while the inset shows the enzyme profiles for the two experiments. Black lines correspond to high and low enzyme controls (see Materials and Methods). Note that there are no measurements between 125 and 175 seconds for the step-function experiment because samples were removed from the fluorimeter (arrow A, 130 sec) so that they could be injected with additional enzyme (arrow B, 150 sec) and re-inserted in the fluorimeter (arrow C, ~170 sec) after mixing. A t-test (t-value = 0.225, p-value = 0.811) confirmed that there was no statistically significant difference between the fluorescence of the two experimental conditions at 300 seconds.

We sought to directly obtain direct experimental confirmation of the validity of our analytical predictions. Towards this goal, we implemented a simple set of assays based on the enzyme β-galactosidase and its synthetic fluorescent substrate 4-methylumbelliferyl β-D-galactopyranoside (MUG). The product of the hydrolysis of MUG by β-galactosidase is fluorescent at 460nm when excited at 360 nm. In one experiment, MUG was exposed to a constant amount of 0.125 U of β-galactosidase for 5 minutes. In a second experiment, the time-profile of β-galactosidase was designed to be a step function, with a concentration of 0.0625U for the first 2.5 minutes, and a concentration of 0.1875 units for an additional 2.5 minutes. Thus, the two assays contained precisely the same average enzyme concentration, but starkly different temporal enzyme profiles. The results of the experiment are shown in Figure 1. As predicted, the concentration of the product (measured by fluorescence) is identical for the two experimental conditions at 5 minutes, i.e. precisely at the moment when their average enzyme levels match.

### Enzyme dynamics as a variational problem

The invariance principle demonstrated above for metabolic reactions indicates that there are infinitely many ways for the cell to modulate enzyme levels to bring substrate from a particular initial concentration to a particular final concentration in a given time interval. The sole common characteristic among these distinct enzyme time courses is the average enzyme level. The next question we ask is whether, given this degeneracy among enzyme time courses, we may expect the cell to use a specific enzyme profile in time. In many cases, cellular regulation of enzyme levels may have evolved so as to perform metabolic tasks with minimal energetic effort, *e.g.* minimal cost of enzyme production and degradation. Hence, we reason that by using appropriate optimization criteria it may be possible to search, among the degenerate enzyme trajectories, for the one that minimizes a given cost function.

What do we expect this cost function to look like? First, notice that we want to pose this question over a time interval (i.e. the time window (*t*_*0*_*, t*_*f*_) ). The total cost *C*_*T*_ to be minimized will therefore be the integral of an instantaneous cost *c*(*t*). This instantaneous cost *c*(*t*) should depend on the shape of the function *E*(*t*). An instantaneous cost with pure linear dependence on *E* would lead to a total cost *C*_*T*_ identical for all trajectories, due to the invariance to average enzyme levels. However, previous work [25] has highlighted the nonlinear nature of individual enzyme cost. This nonlinearity follows from assuming that fitness is a saturating function of common cellular resources, and that these resources are sequestered in proportion to the enzyme concentration. Furthermore, the possible enzyme trajectories may differ significantly in the extent of enzyme production and degradation, which are typically both costly. For example, an average of four high energy phosphate bonds are necessary to add an amino acid to an enzyme being translated [26,27]. Additional energy resources would then need to be devoted to proteases involved in the degradation process [28]. In what follows we will explore how different assumptions on the cost of enzyme production and degradation lead to different optimal enzyme profiles.

Based on the above considerations, we model the cost of an enzyme trajectory by extending the model proposed in [25]. Specifically, we take into account two distinct components which we call the “static” and “dynamic” cost. The static cost is due to the presence of a given amount *E* of total enzyme, which causes the sequestration of enzyme building blocks (resource *R*_*S*_, e.g. amino acids), leading to a nonlinear dependence on *E* (see Materials and Methods). The dynamic cost takes into account the fact that faster transcription-translation (i.e. larger $\dot{E}$) implies increased use of other resources *R*_*D*_ (such as ribosomes). We assume that the dynamic cost is symmetric in its dependence on production and degradation, and hence a function of ${\dot{E}}^{2}$. Furthermore, when the cell is not dedicating any dynamic resources to the maintenance of protein levels, the dynamic cost reaches a minimum. At this minimum, protein levels are only subject to the effects of cellular dilution, which occurs at a rate *μ*. Thus, the dynamic cost should attain a minimum precisely at $\dot{E}=-\mu$, and the dynamic cost should take the form ${\left(\dot{E}+\mu \right)}^{2}$. Upon writing an explicit cost function that combines the static and dynamic components, and performing a Taylor expansion (see Materials and Methods for further details), we obtain an effective instantaneous cost.

(5) $$c\left(t\right)=\overset{\mathrm{Static}\;\mathrm{Cost}}{\overbrace{\alpha_{1}E\left(t\right)+\alpha_{2}E^{2}\left(t\right)}}+\overset{\mathrm{Dynamic}\;\mathrm{Cost}}{\overbrace{\alpha_{3}{\left(\dot{E}\left(t\right)+\mu \right)}^{2}}}\;$$

where the *α* parameters are strictly positive and quantify the relative importance of the static cost to the dynamic cost. Thus, the cost function diverges for very large enzyme levels and production/degradation rates, and achieves a minimum at zero enzyme level and a degradation rate $\dot{E}=-\mu$. While this cost function constitutes an approximation of the full expression, it has the advantage of being analytically tractable (see below), and of capturing the essential nonlinearities in *E* and $\dot{E}$. In the Additional file, we provide details on how to solve the variational problem for a different type of cost function which is asymmetric with respect to $\dot{E}$*.* Importantly, the fitness of an enzyme trajectory would in principle also depend on the *benefit* derived from metabolizing a given amount of substrate with the available enzyme. Here, we focus only on solving the problem of minimizing the cost of an enzyme trajectory for a target average enzyme level (in other words, for a fixed benefit). As discussed in the Conclusion section, the more general problem of maximizing the difference between the benefit and cost of an enzyme trajectory constitutes an interesting open question.

Our problem of cost minimization along enzyme trajectories can be now posed as a variational problem, in which the invariance relative to average enzyme levels plays a key role. Under the constraint that *S*(*0*) *= S*_*0*_, *S*(*t*_*f*_) *= S*_*f*_, we ask what time-course profile *E*(*t*) minimizes the total cost incurred throughout this time interval. This total cost *C*_*T*_ is dependent on the choice of the function *E*(*t*), and can be therefore expressed as a functional:

(6) $$C_{T}\left[E,\dot{E}\right]=\overset{t_{f}}{\underset{t_{0}}{\int }}\left(\alpha_{1}E\left(t\right)+\alpha_{2}E^{2}\left(t\right)+\alpha_{3}{\left(\dot{E}\left(t\right)+\mu \right)}^{2}\right)\mathrm{dt}$$

To solve the problem of minimizing *C*_*T*_, we employ variational calculus. Variational calculus is an optimization technique which finds minima and maxima of functionals. It has had extremely deep impacts in fields such as mechanics (Lagrangian mechanics) and control theory (optimal control), and we refer the reader to [29] for a thorough introduction.

Due to the invariance to average enzyme levels, if we know the appropriate boundary conditions for *S* (*i.e.* how much substrate must be degraded in a certain amount of time), then there must exist a particular average value of enzyme $\bar{E}$ (given *K*_*M*_ and *k*_*2*_) which will satisfy these boundary conditions. Hence, our minimal cost problem can be expressed as

(7) $$\min C_{T}\left[E,\dot{E}\right]\;S.T.\overset{t_{F}}{\underset{t_{0}}{\int }}E\left(t\right)\mathrm{dt}=\;\bar{E}$$

If we let $F=\alpha_{1}E+\alpha_{2}E^{2}+\alpha_{3}{\left(\dot{E}+\mu \right)}^{2}$ and *G* = *E*, then we can pose the optimization problem stated in Eq. (7) in a more familiar form. In particular, minima of this variational problem must satisfy the Euler-Lagrange equation, which takes the form

(8) $$\frac{\mathrm{dF}}{\mathrm{dE}}+\lambda \frac{\mathrm{dG}}{\mathrm{dE}}-\frac{d}{\mathrm{dt}}\left(\frac{\mathrm{dF}}{d\dot{E}}+\lambda \frac{\mathrm{dG}}{d\dot{E}}\right)=0$$

For our particular problem, the Euler-Lagrange equation becomes

(9) $$2\alpha_{3}\ddot{E}=2\alpha_{2}E+\lambda +\alpha_{1}$$

Solving this, we find

(10) $$E\left(t\right)=\frac{-\lambda -\alpha_{1}}{2\alpha_{2}}+C_{1}e^{t\sqrt{\frac{\alpha_{2}}{\alpha_{3}}}}+C_{2}e^{-t\sqrt{\frac{\alpha_{2}}{\alpha_{3}}}}$$

where the constants *C*_*1*_ and *C*_*2*_ and Lagrange multiplier *λ* can be calculated using the boundary conditions and auxiliary constraint:

$$E\left(0\right)=E_{0},E\left(t_{f}\right)=E_{f},\int_{0}^{t_{f}}\mathrm{Edt}=\bar{E}$$

**Figure 2:**
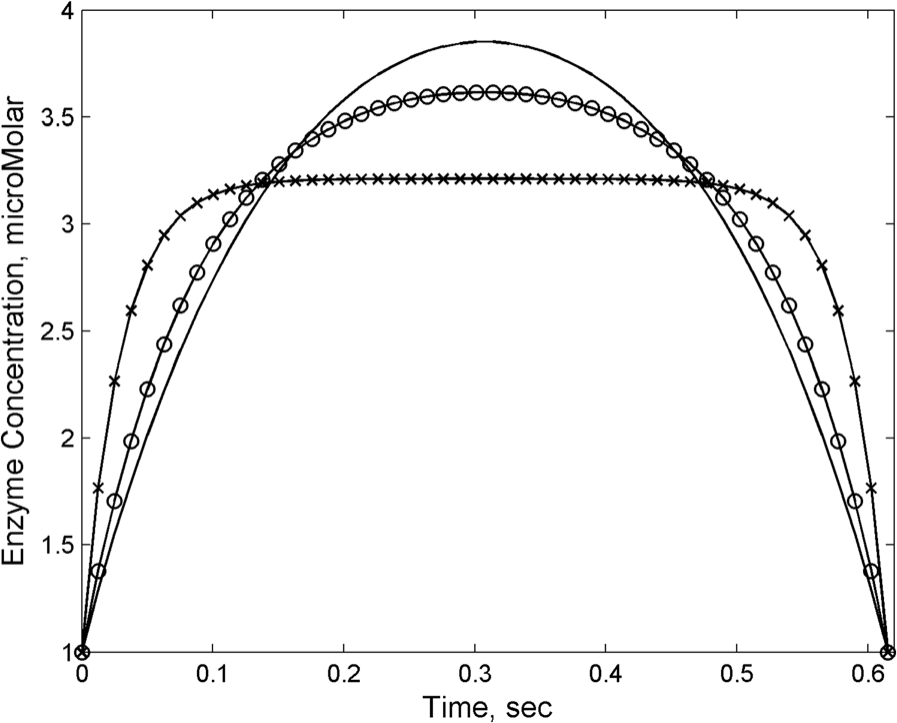
**Optimal enzyme profiles for the cost functional defined in Eq. (10).** The boundary conditions were chosen to be *E*_0_ = *E*_*f*_ = 1 μM, and *Ē* = 3 μM, based on substrate parameters *S*_*0*_*=* 15 μM, *S*_*f*_*=* 2.4 μM*, k*_*2*_=1000 sec^-1^, *k-*_*1*_*=* 1000 sec^-1^, *k*_*1*_*=* 2 μM^-1^sec^-1^, *K*_*M*_ = 1000 μM, *Δt* = 0.6 sec. In inset, different lines correspond to different values of α: solid line (α_1_ = α_2_ = 0.933, α_3_ = .067), circles (α_1_ = α_2_ = 0.981, α_3_ = .019) and crosses (α_1_ = α_2_ = 0.998, α_3_ = .002). For the trajectory with the fastest rate of change of enzyme (α_1_ = α_2_ = 0.998, α_3_ = .002), *δ* = 226, *t*_*E*_*~* .01 sec, *t*_*C*_ = .5 msec.

Eq. (10) provides the enzyme time profile that minimizes the total cost, for a given relative importance of the static versus dynamic cost of protein expression, described by the parameters α_i_. Notably, the ratio of static to dynamic costs depends only on *α*_*2*_/*α*_*3*_, while the magnitude of α_1_ simply rescales the Lagrange multiplier *λ.* Different time-dependent enzyme profiles for different values of *α*_*2*_/*α*_*3*_ are plotted in Figure 2. When *α*_*2*_/*α*_*3*_ becomes large, the cost is entirely static, and the cell pays no price for synthesizing or degrading the enzyme very quickly. At the same time the nonlinear dependence of the cost on *E* penalizes any unnecessary deviation from the average level $\bar{E}$. Hence, as shown in Figure 2, in this case the optimal profile approaches a rectangular shape where enzyme rapidly reaches the necessary average level and remains there for most of the time course. At the opposite extreme (*α*_*2*_/*α*_*3*_ is small, $C_{T}\sim {\dot{E}}^{2}$), there is a substantial cost associated with the production and degradation of enzyme. Under these conditions, the cell will try to make the smoothest transitions possible compatible with the average enzyme constraint, leading to an optimal enzyme profile that approaches a gradual parabolic shape. The transition of enzyme time courses from rectangular profile to parabola reflects the need to balance the costs incurred by using both static and dynamic resources to produce enzyme in the cell.

It should be noted that, for certain choices of parameters, the solution to the variational problem may not satisfy the conditions required for the validity of MM kinetics. There are two specific conditions which need to be verified: (i) that the time scale of *E* is significantly longer than the time scale of *C*, so that the quasi-steady-state assumption used to derive MM kinetics in [20] may still be applied, and (ii) that the concentration of *E* is sufficiently small so that *E << K*_*M*_*+S*. As detailed in the SI Text, one can derive bounds on the enzyme trajectory in Eq. (10) which ensure that these two conditions are both satisfied. While all solutions presented in Figure 2 satisfy these conditions under a realistic choice of parameters, one should be aware, in future work, that optimal solutions of the variational problem may have to be further screened for these criteria.

## Discussion

The metabolic equivalence of enzyme trajectories with identical average enzyme levels Implies that the cell has potentially many ways of achieving a given metabolic goal. At one extreme the strategy is to maintain enzyme level very high, with no need for fast response capabilities. The opposite strategy is to maintain a minimal enzyme level, but be able to respond quickly. All strategies in between these two extremes give rise to the same net metabolic behavior. One may see this spectrum of possibilities as a fundamental tradeoff between the supplies invested in the enzyme itself (static resources) versus the investment necessary for fast enzyme production and degradation (dynamic resources). Such a tradeoff was indeed recently suggested by the analysis of mRNA and protein levels and half-lives in mammalian cells [30]. In particular, it was found that different cellular subsystems exhibited different ratios of resting levels (referred to as "stability") of proteins and mRNAs. For example, enzymes for metabolic subsystems which are frequently used, such as glycolysis, tend to be maintained at high stable levels, compatible with a high usage of static resources, but minimal usage of dynamic ones. Conversely, subsystems which are less frequently used but must react quickly tend to have stable mRNA but unstable proteins, consistent with low sequestration of static resources but a high demand on dynamic resources.

Looking forward, a prospect for future studies will be assessing whether the invariance to average enzyme levels may be generalized to biological conditions *in vivo*. We suggest that two approaches may be taken toward addressing this question. First, current high-throughput technologies are beginning to enable the quantification of temporal variation in enzyme abundance arising naturally within the cell. Changes in abundance are affected by a variety of distinct processes, from phosphorylation and dephosphorylation cycles to enzyme translation and degradation. By concurrently monitoring the amount of metabolite converted by a reaction and the dynamical changes of the corresponding active enzyme pool, it may be possible to assess the validity of our findings in a living system. A second approach may arise from newly developed techniques in synthetic biology, where the expression of a single enzyme may be precisely and arbitrarily controlled over time. In particular, in analogy with the previously published analysis of yeast’s response to a nutrient supply “signal” that oscillates at a given frequency [31], it may be possible to study the input–output properties of reactions and pathways based on signal processing techniques. For example, one could explore whether the insensitivity to certain enzyme changes demonstrated here could be explained in terms of a low pass filter. Indeed, such behavior was observed in [31], where very fast variations in periodic changes of the carbon source were ignored by the yeast population.

Our analysis highlights the existence of a spectrum of resource utilization strategies and ensuing tradeoffs in enzyme dynamics. It will be interesting to more directly assess whether the choice of specific strategies for metabolic regulation could be a direct target of evolutionary adaptation. As pointed out in [30], mRNA and protein levels for different subsystems may be explainable as optimal strategies for energy usage, specifically targeted by different evolutionary pressures. The invariance to average enzyme levels allows one to address this question in a general way, namely by asking what enzyme profile minimizes a given combination of static and dynamic enzyme-associated cost. For certain categories of cost functions, the problem can be solved analytically, using variational calculus. We focused specifically on the case of a cost function that is inspired by a previously experimentally validated cost-benefit model [25]. In this case, the optimality principle provides specific enzyme trajectories whose dependence on enzymatic parameters is amenable to future experimental testing. Importantly, the timescales of enzyme change predicted by the variational principle may overlap with timescales of substrate kinetics, suggesting that our findings should be interpreted under the assumption that enzyme changes may result from a combination of slow (e.g., transcription-translation and degradation) and fast (e.g., allosteric regulation, or phosphorylation) processes.

The use of symmetry principles and variational calculus in physics is widespread and has led to very important conceptual and practical insight. Some progress has been made in applying similar variational principles to biological problems, usually in the framework of optimal control theory using numerical optimizations (for example, [21,32]). It will be interesting to see whether variational approaches like the one we are proposing can be used to rederive some of the results from [21,32] analytically. Variational calculus has also been applied to metabolism in a very different context, namely the study of thermodynamic constraints on metabolic fluxes [33].

## Conclusions

A major open challenge in systems biology is to model metabolic and regulatory networks in a unified mathematical framework. At present, there is a tendency to develop models for larger-scale and more detailed representations of cellular networks, whose complex dynamic behavior is measurable with high throughput experiments. In contrast, we focus here on enzyme regulation for a single metabolic reaction and suggest that new insight can still be obtained by studying this fundamental building block of biochemical networks, the “hydrogen atom” of metabolism. Despite the apparent complexity of dealing with time-dependent enzyme concentrations, we show that for a category of problems there is an underlying simplicity. The fact is that, in simple metabolic reactions, different enzyme trajectories with identical average levels yield the same substrate flux over a given time interval. A standard enzyme assay allowed us to experimentally confirm the validity of this result.

Given the potential insight into metabolic regulation from invariance laws and variational calculus, we conclude by posing two challenges for future research, whose resolution would substantially increase the impact of this approach.

*First*, suppose that the benefit that the cell obtains from metabolizing a given amount of substrate, Δ*S*, in a time interval, Δ*t*, can be described by a function *B* = *B*(Δ*S*, *E*, Δ*t*). Then, extending previous work (e.g. [21,25,34]) one could try and solve through variational calculus the biologically more accurate problem of maximizing the difference between such benefit, and the enzyme-associated cost described by Eq. (6). Under reasonable assumptions on *B, i.e.*$B\;\left(i.e.\frac{\partial B}{\partial \mathrm{\Delta S}}>0,\frac{\partial^{2}B}{\partial \Delta S^{2}}<0\right)$, do general strategies exist for how the cell should modulate enzyme levels in order to maximize the difference between benefit and cost? If so, what are the characteristics of these strategies?

*Second*, the relevance of our variational approach to deeper biological questions is contingent on the feasibility of extending this formalism to larger metabolic systems. In particular, is it possible to find invariant quantities, relating enzyme levels to net changes in substrate concentrations, for systems comprised of more than one reaction, i.e. metabolic networks? Can such invariant quantities be exploited to understand strategies for the regulation of whole pathways or even larger modules?

Irrespective of whether these questions can be solved analytically, thinking about metabolic regulation in terms of dynamical optimization of trajectories may pave new ways of understanding how living systems gather their regulatory machinery to respond to environmental changes, and in turn guide synthetic biology efforts for the design of engineered organisms.

## Methods

### Enzyme Assay

For the enzyme assay, 6.9 mg of 4-methylumbelliferyl β-D-galactopyranoside (MUG) was diluted in 50 mL of buffer containing 100 mM sodium phosphate, 1 mM MgCl_2_, and 50 mM beta-mercaptoethanol. β-galactosidase dilutions were made fresh daily from a stock of 250 U/mL with deionized water to 2.5 U/mL. Enzyme was kept on ice during use. Fluorescence (corresponding to the concentration of MUG) was monitored every second over the length of the study, by excitation at 360 nm and measuring emission at 460 nm. Samples were maintained at 37°C during measurements. In the first experiment, 50 μL of β-galactosidase were added to 3 mL of MUG solution and monitored for 5 minutes for fluorescence. For the second experiment, 25 μL of β-galactosidase were added to 3mL of MUG solution and fluorescently monitored for 2.5 minutes, followed by an additional injection of 50 μL of β-galactosidase, and again monitored for 2.5 minutes. The net difference in working volume between the two experimental conditions was 25 μL out of 3 mL, or approximately 1%, and was deemed to be negligible in contrast to other sources of error in the system. Two controls were used for the experiment. In the high enzyme control, 75 μL of β-galactosidase were added to 3 mL of MUG solution and monitored for 5 minutes. In the low enzyme control, 25 μL of β-galactosidase were added to 3 mL of MUG solution and monitored for 5 minutes.

All the enzyme used in the experiment was prepared ahead of time, and placed in ice to prevent denaturation. We performed each of the nine replicates in the experiment (3 replicates for each time course × 3 time courses = 9 total replicates) one after the other. The total time for the experiments was approximately two hours, and in this time we observed negligible loss of catalytic activity.

### Derivation of instantaneous enzyme cost function

To identify, among all possible equivalent enzyme time courses, the ones of potential biological significance, we need to estimate the cost associated with each trajectory. We model the cost of a trajectory by extending the model proposed in [25]. Specifically, we assume that the cost a cell incurs upon producing an enzyme can be calculated as the reduction in the cell’s fitness due to the utilization and sequestration of resources. Extending the previous model, we take into account two distinct components of fitness which we call the “static” and “dynamic” fitness (associated with resources *R*_*S*_ and *R*_*D*_ respectively). We assume a symmetric cost of both production $\left(\dot{E}>0\right)$ and degradation $\left(\dot{E}<0\right)$ of enzyme, so that the dynamic cost is dependent on ${\dot{E}}^{2}$. Furthermore, cellular growth results in the dilution of cellular resources at a rate *μ.* Thus, the minimum dynamic cost (at which the cell is not expending dynamic resources) occurs when $\dot{E}=-\mu$. Then, following [25], the total fitness in the presence of enzyme level *E* and enzyme change rate $\dot{E}$ is expressed as

(11) $$f\left(E,\dot{E}\right)=\frac{R_{S}-E}{K+R_{S}-E}∙\frac{R_{D}-{\left(\dot{E}+\mu \right)}^{2}}{K+R_{D}-{\left(\dot{E}+\mu \right)}^{2}}$$

The cost can then be computed as

(12) $$\begin{array}{ll} c\left(E,\dot{E}\right) & =\frac{f\left(E,\dot{E}\right)-f\left(0,0\right)}{f\left(0,0\right)}\\ & =\frac{C_{1}E+C_{2}{\left(\dot{E}+\mu \right)}^{2}+C_{3}E{\left(\dot{E}+\mu \right)}^{2}}{\left(C_{4}-C_{5}E\right)\left(C_{6}-C_{7}{\left(\dot{E}+\mu \right)}^{2}\right)} \end{array}$$

where the *C*_*i*_ parameters can be inferred from Eq. (11). The resulting expression in Eq. (12) is not directly amenable to analytical calculations. However, we can capture the main nonlinearity of this cost function (such as its linear dependence with respect to *E* when is *E* is sufficiently small, and its rapid divergence when *E* is large) in a simplified version, obtainable through a Taylor expansion about zero. In particular, we assume the sequestration of static and dynamic resources to be small in comparison to the total amount of resources in the cell (*i.e.* both *E* and ${\left(\dot{E}+\mu \right)}^{2}$ are sufficiently small so that the *E* and ${\left(\dot{E}+\mu \right)}^{2}$ terms dominate over other terms in a Taylor expansion), and that the coupling between static and dynamic costs (captured by the $C_{3}E{\left(\dot{E}+\mu \right)}^{2}$ term in the numerator of Eq. (12)) is sufficiently small so as to be negligible. This yields the instantaneous cost $c\left(t\right)=\alpha_{1}E\left(t\right)+\alpha_{2}E^{2}\left(t\right)+\alpha_{3}{\left(\dot{E}\left(t\right)+\mu \right)}^{2}$, where the *α*_*i*_ are positive constants. More accurate approximations of Eq. (12), such as those which account for the coupling between static and dynamic resources, are possible, but may lead to analytically intractable dynamic optimization problems.

## Reviewer comments

### Reviewer 1: Dr. Sergei Maslov

Comment:*I liked the conceptual idea of this study according to which organisms are solving a dynamical optimization problem that in addition to static terms introduced in Ref.*[25]*also includes dynamic terms. These terms quantify energy and sequestration costs of operating ribosomes as well as other (smaller) costs associated with protein production and active degradation. I also liked the fact that authors confirmed their mathematical results with a dedicated experimental data.*

Response: We are grateful to the reviewer for the positive feedback, and very helpful comments, which we address in detail below.

Comment:*I found the application of this conceptual idea somewhat less convincing. Figure*2*shows an optimal enzyme profiles for different relative weights of static and dynamic terms. The biological relevance of the optimization problem solved in this study is not well explained. Indeed, why should an organism care to find the best E(t) for given <E> and S_f/S ? Should not it be that both S_f/S as well as E(t) need to be optimized to get the best fitness? I would be much more interested if authors ’formalism could quantify what they stated in the discussion section: “… enzymes for metabolic subsystems which are frequently used, such as glycolysis, tend to be maintained at high stable levels, compatible with a high usage of static resources, but minimal usage of dynamic ones. Conversely, subsystems which are less frequently used but must react quickly tend to have stable mRNA but unstable proteins, consistent with low sequestration of static resources but a high demand on dynamic resources.” To quantify this tradeoff authors need to apply their optimization formalism to multiple (periodic?) cycles of nutrient availability as opposed to a single cycle considered in this study.*

Response: We thank the reviewer for giving us the opportunity to clarify and elaborate on the biological relevance of the cost functional we use in the variational problem. We addressed this issue in multiple ways:

First, we moved the explicit derivation of our cost function from the Additional file 1 SI to the Materials and Methods section. This derivation, now expanded for increased clarity, shows in detail how we arrive at our cost functional based on previously proposed models of protein cost, and on simplifying assumptions.

Second, in presenting the cost function, we now explicitly mention that fitness of the cell would in principle depend both on the cost of enzyme production, as well as on the benefit derived from metabolized a given amount of substrate (in between Eqs. (5) and (6)). However, the solution of an optimization problem using a cost functional (a function of functions of time) is quite different, and substantially harder to solve, than previous optimization problems using cost functions, which depended simply on two variables (such as substrate and enzyme levels in [25]). Our approach is in itself a novel way of expressing a problem of metabolic regulation, making it possible to use variational calculus for obtaining exact analytical solutions.

Third, as far as we can tell, the only way to explicitly account for the benefit derived from metabolizing substrate using our current framework (other than solving a much more complicated and potentially intractable variational problem with two dependent functions E(t), and S(t) ) would be to invert Eq. (4), and express substrate consumed as a function of <E>, using the Lambert W function. Doing so renders the variational problem, which now contains the W function in the objective, analytically intractable. However, the reviewer raises a very compelling and challenging question, which we now pose as an open problem for future research at the end of our Conclusion section. Notably, a special case of our proposed problem would be the situation where a substrate is subject to multiple, periodic inflows over a time interval.

Comment:*For a constant growth rate \mu maintaining E_dot =0 requires constant production to compensate for dilution term. Thus E_dot=0 is not the minimum and the minimum should be reached at E_dot=-\mu. Would that modify authors’conclusions?*

Response: We thank the reviewer for the insightful point. Indeed, when the cell is not dedicating any dynamic resources to maintaining protein levels, these levels should experience a dilution effect. We have accounted for this effect in our calculations in the main text, as shown in Eqs. (5) and (6). This modification does not modify the outcome of the optimization problem, or our conclusions.

Comment:*Dimensionality of Eq. (6) is confusing. Apparently E is made dimensionless so that E+E^2 makes sense and time is also made dimensionless. These normalizations should be better explained. Perhaps they contain some information on the typical value of alpha.*

Response: We thank the reviewer for pointing out this problem with dimensions in the definition of the cost function. Indeed, as suggested by the reviewer, we could have resolved this by defining non-dimensional variables. Instead, we revised our use of the coefficients in the definition of the cost (Eq. (5)) to make it clear that these constants have distinct dimensions, so that the terms of the cost function are consistent with each other dimensionally. We have revised our calculations throughout the manuscript, including Figure 2, to reflect this point.

Comment:*For the sake of completeness the expression for K_M in terms of k_1 and k_{-1} and k_2 needs to be written.*

Response: Thank you for the suggestion. We have explicitly indicated immediately following Eq. (2) the relationship between *K*_*M*_ and the underlying mass-action rate constants.

Comment:*Use brackets under the integral in Eq. (6).*

Response: Thank you for the comment. We have modified the notation in Eq. (6) in line with the reviewer’s recommendation.

### Reviewer 2: Dr. William Hlavacek

Comment:*The authors consider a system in which the level of an enzyme can vary with time. The kinetics of the reaction catalyzed by the enzyme E+S<->(ES)->E+P is assumed to be governed by the Michaelis-Menten rate law. The authors show that the amount of substrate consumed over a given period of time depends only on the average level of enzyme. The derivation of this result is straightforward. I have not seen the result discussed before. It is conceivable that the result is already in the literature, somewhere. In any case, it is probably fair to say that the result is not well known. The authors validate the result experimentally, which is interesting. Because there are many trajectories E(t) that yield the same average enzyme level, the authors investigate optimality. For certain assumptions and a cost function taken from the literature, the authors solve an optimization problem and derive a formula for the optimal time-dependent enzyme level. The cost function could be explained in a little bit more detail and more clearly in the manuscript, although it is not absolutely necessary, because a reference is given. The authors are careful to think about and/or check various assumptions upon which their derivations are based and to show how the analysis extends to rate laws more complex than the Michaelis-Menten rate law. The manuscript is well written, and the work appears to be technically sound. The report could be useful to researchers who are interested in how time-dependent enzyme levels (varying because of gene regulation) influence metabolic reactions.*

Response: We are grateful to the reviewer for the positive feedback and useful comments. We were indeed surprised ourselves not to find the dependence on the average enzyme anywhere in the literature, not even as a simple exercise in textbooks. To reflect the reviewer’s suggestion that it may have been written somewhere before, we modified accordingly the paragraph immediately preceding the section on Experimental Validation. We also added a new sentence to clarify the definition of the cost function, right after its definition. In addition, we now mention more explicitly that a detailed derivation and additional comments on the biological significance of this cost function are presented in the Supplementary Materials.

Comment:*A possible nice way to improve the manuscript, which might increase its potential impact a little bit, would be to extend the analysis to consider a (small) metabolic network, meaning a system with more than one reaction.*

Response: We thank the reviewer for pointing to this interesting challenge, which we had ourselves wondered about. Our ability to derive analytical results for this system largely depended on relating changes in substrate levels to average enzyme levels via an invariance principle. It remains unclear to us whether such invariance principles exist for larger, more complicated metabolic systems. In light of this comment, we have added at the end of our Conclusion section an explicit mention of this idea, and of the associated mathematical challenge. By making the community aware of the difficulty of this problem and of the potential insight to be gained (i.e. the possibility of understanding metabolic regulation through variational calculus), we hope to catalyze a route to its resolution.

### Reviewer 3: Dr. Daniel Kahn

This reviewer provided no comments for publication.

## Competing interests

The authors declare that they have no competing interests.

## Authors’ contributions

ER and DS conceived the study, completed the analysis, and wrote the manuscript. SY and ER performed the experiment. ER, SY and DS read and approved the final manuscript.

## Supplementary Material

Additional file 1 SI Text: Supplementary information referenced in main text. Click here for file

## References

[B1] van Rijsewijk BRB H Nanchen A Nallet S Kleijn RJ Sauer U Large-scale 13C-flux analysis reveals distinct transcriptional control of respiratory and fermentative metabolism in Escherichia coli Mol Syst Biol 2011 7 477 21451587 10.1038/msb.2011.9 PMC3094070

[B2] Youk H Van Oudenaarden A Growth landscape formed by perception and import of glucose in yeast Nature 2009 462 875 879 10.1038/nature08653 20016593 PMC2796206

[B3] Grüning N-M Lehrach H Ralser M Regulatory crosstalk of the metabolic network Trends Biochem Sci 2010 35 220 227 10.1016/j.tibs.2009.12.001 20060301

[B4] Fendt S-M Buescher JM Rudroff F Picotti P Zamboni N Sauer U Tradeoff between enzyme and metabolite efficiency maintains metabolic homeostasis upon perturbations in enzyme capacity Mol Syst Biol 2010 6 356 20393576 10.1038/msb.2010.11 PMC2872607

[B5] Orth JD Thiele I Palsson BØ What is flux balance analysis? Nat Biotechnol 2010 28 245 248 10.1038/nbt.1614 20212490 PMC3108565

[B6] Lewis NE Nagarajan H Palsson BO Constraining the metabolic genotype-phenotype relationship using a phylogeny of in silico methods Nat Rev Microbiol 2012 10 291 305 22367118 10.1038/nrmicro2737 PMC3536058

[B7] Piedrafita G Montero F Morán F Cárdenas ML Cornish-Bowden A A simple self-maintaining metabolic system: robustness, autocatalysis, bistability PLoS Comput Biol 2010 6 9 10.1371/journal.pcbi.1000872 PMC2916848 20700491

[B8] Frieden C Slow transitions and hysteretic behavior in enzymes Annu Rev Biochem 1979 48 471 489 10.1146/annurev.bi.48.070179.002351 382990

[B9] Frieden C Kinetic Aspects of Regulation of Metabolic Processes. The hysteretic enzyme concept J Biol Chem 1970 245 5788 5799 5472372

[B10] Laget PP Hysteretic properties of soluble F1 ATPase from Escherichia coli. II. Nucleotide effects on the slow changes of the enzyme kinetic behaviour. Arch Biochem Biophys 1979 192 474 481 10.1016/0003-9861(79)90117-6 155423

[B11] Roussel M Slowly reverting enzyme inactivation: a mechanism for generating long-lived damped oscillations J Theor Biol 1998 195 233 244 10.1006/jtbi.1998.0788 9822565

[B12] Roussel MR The use of delay differential equations in chemical kinetics J Phys Chem 1996 100 8323 8330 10.1021/jp9600672

[B13] Schnell S Hanson SM A test for measuring the effects of enzyme inactivation Biophys Chem 2007 125 269 274 10.1016/j.bpc.2006.08.010 17011111

[B14] Waley SG Kinetics of suicide substrates Biochem J 1980 185 771 773 7387634 10.1042/bj1850771 PMC1161457

[B15] Cornish-Bowden A Fundamentals of Enzyme Kinetics 2012 Weinheim: Wiley-Blackwell

[B16] Collins SB Reznik E Segrè D Temporal expression-based analysis of metabolism PLoS Comput Biol 2012 8 11 e1002781 10.1371/journal.pcbi.1002781 23209390 PMC3510039

[B17] Reznik E Kaper TJ Segrè D The dynamics of hybrid metabolic-genetic oscillators Chaos 2013 23 013132 10.1063/1.4793573 23556969

[B18] Karr JR Sanghvi JC Macklin DN Gutschow MV Jacobs JM Bolival B Assad-Garcia N Glass JI Covert MW A whole-cell computational model predicts phenotype from genotype Cell 2012 150 389 401 10.1016/j.cell.2012.05.044 22817898 PMC3413483

[B19] Smallbone K Simeonidis E Swainston N Mendes P Towards a genome-scale kinetic model of cellular metabolism BMC Syst Biol 2010 4 6 10.1186/1752-0509-4-6 20109182 PMC2829494

[B20] Segel LA Slemrod M The quasi-steady-state assumption: a case study in perturbation SIAM Rev 1989 31 446 10.1137/1031091

[B21] Wessely F Bartl M Guthke R Li P Schuster S Kaleta C Optimal regulatory strategies for metabolic pathways in Escherichia coli depending on protein costs Mol Syst Biol 2011 7 155 10.1038/msb.2011.46 PMC3159982 21772263

[B22] Fung E Wong WW Suen JK Bulter T Lee S Liao JC A synthetic gene-metabolic oscillator Nature 2005 435 118 122 10.1038/nature03508 15875027

[B23] Yuan J Doucette CD Fowler WU Feng X-J Piazza M Rabitz HA Wingreen NS Rabinowitz JD Metabolomics-driven quantitative analysis of ammonia assimilation in E. coli Mol Syst Biol 2009 5 302 19690571 10.1038/msb.2009.60 PMC2736657

[B24] Rondelez Y Competition for catalytic resources alters biological network dynamics Phys Rev Lett 2012 108 018102 22304295 10.1103/PhysRevLett.108.018102

[B25] Dekel E Alon U Optimality and evolutionary tuning of the expression level of a protein Nature 2005 436 588 592 10.1038/nature03842 16049495

[B26] Nelson DL Cox MM Freeman WH Lehninger Principles of Biochemistry, Fourth Edition [Hardcover] 2004 4 1100

[B27] Wagner A Energy constraints on the evolution of gene expression Mol Biol Evol 2005 22 1365 1374 10.1093/molbev/msi126 15758206

[B28] Kenniston JA Baker TA Fernandez JM Sauer RT Linkage between ATP consumption and mechanical unfolding during the protein processing reactions of an AAA+ degradation machine Cell 2003 114 511 520 10.1016/S0092-8674(03)00612-3 12941278

[B29] Gelfand IM Fomin SV Calculus of Variations 2000 Mineola: Dover Publications 240

[B30] Schwanhäusser B Busse D Li N Dittmar G Schuchhardt J Wolf J Chen W Selbach M Global quantification of mammalian gene expression control Nature 2011 473 337 342 10.1038/nature10098 21593866

[B31] Bennett MR Pang WL Ostroff NA Baumgartner BL Nayak S Tsimring LS Hasty J Metabolic gene regulation in a dynamically changing environment Nature 2008 454 1119 1122 10.1038/nature07211 18668041 PMC2654342

[B32] Bartl M Li P Schuster S Modelling the optimal timing in metabolic pathway activation-use of Pontryagin’s Maximum Principle and role of the Golden section Bio Systems 2010 101 67 77 10.1016/j.biosystems.2010.04.007 20420882

[B33] Fleming RMT Maes CM Saunders MA Ye Y Palsson BØ A variational principle for computing nonequilibrium fluxes and potentials in genome-scale biochemical networks J Theor Biol 2012 292 71 77 21983269 10.1016/j.jtbi.2011.09.029

[B34] Chiu H-C Marx CJ Segrè D Epistasis from functional dependence of fitness on underlying traits Proc Biol Sci Roy Soc 2012 279 4156 4164 10.1098/rspb.2012.1449 PMC3441082 22896647

