# Supplementary material for: Invariance and optimality in the regulation of an enzyme
Source: Biol Direct. 2013 Mar 22;8:7. doi: 10.1186/1745-6150-8-7 (PMC3665469; doi:10.1186/1745-6150-8-7)
Supplement: Additional file 1 — SI Text: Supplementary information referenced in main text. [file 1745-6150-8-7-S1.docx]

**Supporting Information**

*Extending the invariance principle to more complex biochemical reactions*

Consider the general form for the kinetics of a reaction with two substrates *S1* and *S2* [1].

|  | $\frac{dS_{1}}{dt}=\frac{dS_{2}}{dt}=\frac{-k_{2}E\left( t \right)S_{1}S_{2}}{K_{1}+K_{2}S_{1}+K_{3}S_{2}+S_{1}S_{2}}$ |  |
| --- | --- | --- |

We would like to derive a relation similar to Eq. (4) which relates average enzyme levels to substrate flux. The problem becomes more tractable if we make the change variables *S = S1+S2* and *D = S­1-S2*. Then, *S* represents the sum of the concentrations of the two metabolites and *D* the difference. Note that although *S* changes over time, *D* is fixed (in other words, the magnitude of the difference between the two metabolite concentrations remains fixed over the course of the entire reaction). Then, we can rewrite Eq. (S1) as

|  | $\frac{dS}{dt}=-\frac{k_{2}E\left( t \right)\left( S^{2}-D^{2} \right)}{2K_{1}+\left( K_{2}+K_{3} \right)S+\left( K_{2}-K_{3} \right)D+\left( S^{2}-D^{2} \right)}$ |  |
| --- | --- | --- |

We can separate variables as before and after careful algebra find that

|  | $S_{f}-S_{0}+\frac{1}{2}C_{2}\ln\frac{S_{f}^{2}-D^{2}}{S_{0}^{2}-D^{2}}-\frac{C_{3}+D^{2}}{2D}\ln\left( \frac{\left( S_{f}+D \right)\left( S_{0}-D \right)}{\left( S_{0}+D \right)\left( S_{f}-D \right)} \right)=-2k_{2}\bar{E}\Delta t$ |  |
| --- | --- | --- |

Next, we considered a case where two metabolites compete for the active site of an enzyme molecule. We modeled such a system using the equations

|  | $\frac{dS_{1}}{dt}=\frac{-k_{2}E\left( t \right)S_{1}}{K_{M}+S_{1}+S_{2}}$ |  |
| --- | --- | --- |

|  | $\frac{dS_{2}}{dt}=\frac{-k_{2}E\left( t \right)S_{2}}{K_{M}+S_{1}+S_{2}}$ |  |
| --- | --- | --- |

We then made a change of variables and modeled the dynamics of the total ammount of substrate *ST*=*S1+S2*, which is governed by the much simpler differential equation

|  | $\frac{dS_{T}}{dt}=\frac{-k_{2}E\left( t \right)S_{T}}{K_{M}+S_{T}}$ |  |
| --- | --- | --- |

This equation is identical to Eq. (2), and thus exhibits an identical invariance to average enzyme levels, except now the *total* amount of substrate degraded is invariant to enzyme dynamics. In order to calculate the concentrations of the individual substrate molecules *S1* and *S2*, note that

|  | $\frac{\frac{dS_{1}}{dt}}{\frac{dS_{2}}{dt}}=\frac{dS_{1}}{dS_{2}}=\frac{S_{1}}{S_{2}}$ |  |
| --- | --- | --- |

Solving this differential equation with the initial conditions $S_{1}\left( 0 \right)=S_{1}^{0}, S_{2}\left( 0 \right)=S_{2}^{0}$, we find that $S_{1}\left( t \right)=\frac{S_{1}^{0}}{S_{2}^{0}}\cdot S_{2}\left( t \right)$, *i.e.* that their ratio remains constant for all time.

*Solution of an alternative variational problem*

While the cost functional Eq. (6) describes the case when enzymatic production and degradation are penalized equally, nature may in many cases dictate an enzyme cost function which is asymmetric in some way. Such cost functions are still tractable using the same analytical techniques as before. We present such a candidate cost function here. Consider the case of an assymetric cost which penalizes only dynamic resource sequestration, and does so in a way that penalizes enzyme production more than enzyme degradation. A reasonable cost functional would then be

|  | $C_{T}[E,\dot{E}]=\int_{t_{0}}^{t_{f}} \left( e^{\dot{E}+\mu}-\dot{E}+\mu\right)dt$ |  |
| --- | --- | --- |

Applying the invariance to average enzyme levels, we now have a minimization problem of the form

|  | $\min\int_{t_{0}}^{t_{f}} \left( e^{\dot{E}+\mu}-\dot{E}+\mu\right)dt S.T. \int_{t_{0}}^{t_{f}} Edt=\bar{E}\Delta t$ |  |
| --- | --- | --- |

Again writing the E-L equation, we arrive at the second order differential equation

|  | $\ddot{E}e^{\dot{E}+\mu}=\lambda$ |  |
| --- | --- | --- |

This can be solved to find the solution

|  | $E\left( t \right)=\left( \frac{C}{\lambda}+t \right)\ln\left( C+\lambda t \right)-(\mu+1)t+D$ |  |
| --- | --- | --- |

where the constants and Lagrange multiplier are again determined by boundary conditions and the auxiliary constraint.

*Criteria for Validity of Optimal Trajectories*

It should be noted that, for certain choices of parameters, the solution to the variational problem may not satisfy our earlier assumptions required to generate time-dependent MM kinetics. There are two specific conditions which need to be verified: (i) that the time scale of *E* is significantly longer than the time scale of *C*, and (ii) that the abundance of *E* is sufficiently small. With regard to the first condition, this could in principle happen as *α* approaches one, since the shape of *E*(*t*) becomes significantly steeper (because the optimal solution calls for higher rates of change of enzyme). One way to confirm that *E* does not change too fast is to ensure that the time scale of *E* is much longer than the time-scale of *C* calculated in [2]: *tC=1/k1*(*S+KM*). We can make a conservative estimate regarding the time scale of *E*, *tE*, by noting that the maximal rate of change of *E* in Eq.(14) occurs at the boundaries of the solution *t0* and *tf .* Then, following [2] , we can estimate *tE* by $E_{0}/\left|\frac{dE}{dt}\right|_{\max}$. Writing the condition relating the time scales of *E* and *C* explicitly, we find

|  | $\frac{E_{0}}{\left\vert\frac{dE}{dt} \right\vert_{\max}}=\frac{E_{0}}{\sqrt{\alpha_{2}/\alpha_{3}}\left( C_{1}-C_{2} \right)}\gg\frac{1}{k_{1}\left( S_{f}+K_{M} \right)}$ |  |
| --- | --- | --- |

The denominator of the rightmost term in Eq. (S14) is written in terms of the boundary value *Sf* because this corresponds to our most conservative estimate on *tC.* Each of the trajectories in Fig. 2 satisfy condition (S12).

The second condition described above ensures that solutions which call for very high average enzyme levels will not invalidate our earlier assumption that *E << KM + S*. Assuming that the variation in *E* is not too large, a reasonable way to assure that this condition is satisfied is to ensure that the average enzyme required for a particular flux is not too high, i.e.:

|  | $\bar{E}\ll K_{M}+S_{f}$ |  |
| --- | --- | --- |

We again use the boundary value for *S* in order to obtain the most conservative bound on $\bar{E}$. Substituting Eq. (4) into Eq. (S13) and rearranging algebraically to isolate *Sf*, we can derive the condition that

|  | $\delta=\frac{k_{2}\Delta t\left( 1+\frac{S_{f}}{K_{M}} \right)+\frac{S_{f}}{K_{M}}+\ln S_{f}}{\ln S_{0}+\frac{S_{0}}{K_{M}}}\gg1$ |  |
| --- | --- | --- |

where *δ* is a nondimensional quantity. Qualitatively, Equation (S14) gives us a bound on the smallest final substrate concentration for which we can expect that the required average enzyme level is still sufficiently small. Note that the condition in Equation (S14) may not be valid if the maximal value of *E*(*t*), *Emax,* is significantly larger than $\bar{E}$. In this case, *Emax*, will replace $\bar{E}$in Equation (S13). The trajectories in Fig. 2 satisfy condition (S14).

1. Fell DA: **Metabolic control analysis: a survey of its theoretical and experimental development.** *The Biochemical journal* 1992, **286 ( Pt 2**:313–30.

2. Segel LA, Slemrod M: **The Quasi-Steady-State Assumption: A Case Study in Perturbation**. *SIAM Review* 1989, **31**:446.
